# Inhibiting responses under the watch of a recently synchronized peer increases self-monitoring: evidence from functional near-infrared spectroscopy

**DOI:** 10.1098/rsob.230382

**Published:** 2024-02-21

**Authors:** R. Moffat, N. Caruana, E. S. Cross

**Affiliations:** ^1^ School of Psychological Sciences, Macquarie University, Sydney, New South Wales 2109, Australia; ^2^ MARCS Institute for Brain, Behaviour and Development, Western Sydney University, Westmead Innovation Quarter Building U, Westmead New South Wales 2145, Australia; ^3^ Institute of Neuroscience and Psychology, University of Glasgow, Glasgow G12 8QB, UK; ^4^ Professorship for Social Brain Sciences, ETH Zurich, Zurich 8092, Switzerland; ^5^ College of Education, Psychology and Social Work, Flinders University, Bedford Park, South Australia, Australia

**Keywords:** inhibitory control, motor synchrony, interpersonal coordination, social alignment, functional near-infrared spectroscopy, self-monitoring

## Abstract

Developing motor synchrony with a peer (through interventions such as the mirror game) can yield collaborative, cognitive and social benefits. However, it is also well established that observation by an audience can improve cognition. The combined and relative advantages offered by motor synchronization and audience effects are not yet understood. It is important to address this gap to determine the extent to which synchronizing activities might interact with the positive effects of an audience. In this preregistered study, we investigate the extent to which response inhibition may be improved when observed by a peer after motor synchronization with this peer. We compare behavioural and cortical (functional near-infrared spectroscopy; fNIRS) measures of inhibition between synchronized and non-synchronized dyads and find that the presence of a synchronized peer-audience introduces a speed–accuracy trade-off, consisting of slower reaction times and improved accuracy. This co-occurs with cortical activation in bilateral inferior frontal and middle prefrontal cortices, which are implicated in monitoring and maintenance of social alignment. Our findings have implications for carers and support people, who may benefit from synchronizing activities for rehabilitating inhibition and social skills in clinical settings.

## Graphical abstract

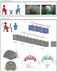

## 1. Introduction

Motor synchrony, the alignment of bodily movements in space and time, has been shown to act as a form of ‘social glue’ that supports communication, collaboration and prosocial behaviour, as well as enhancing our perceptions of the people we interact with and our subjective experiences during these interactions [1–4]. A growing number of studies demonstrate that motor synchrony interventions can improve aspects of social cognition, such as joint attention and social mimicry [5–8]. Recently, studies demonstrated that motor synchrony that takes place between participants and trained confederates may also enhance cognition more generally [9–12]. Specifically, Rauchbauer *et al*. [12] showed that such motor synchrony interventions can lead to improved automatic imitation inhibition, while Keisari *et al*. [9] demonstrated the positive impacts of such interventions on working-memory and attentional function. Both studies assessed participants' cognitive performance after the synchronized partner (i.e. a confederate) left the room, offering insight into the impact of induced motor synchrony on subsequent cognitive performance, but neither study shed light on whether the continued presence or attention of the synchronized partner influences cognitive performance. However, given that motor synchrony is most likely to emerge during sustained interactions, it is also pertinent to understand how cognition is influenced in the presence of a synchronized social partner. Thus, an important question remains concerning how the presence of a partner, with whom one has recently synchronized, influences cognitive performance. Drawing on the wealth of empirical evidence that an audience—even a single peer—can lead to improved cognitive performance [13], we conducted a preregistered investigation to explore the extent to which a synchronized audience improves cognitive performance more than a non-synchronized audience.

In the following sections, we first discuss motor synchrony and social connectedness, then review audience effects on cognitive performance. Next, we present the rationale for investigating how combining a motor synchronization activity with a partner, who subsequently becomes an ‘audience’, could lead to improved response inhibition, before proceeding to detail our hypotheses.

### 1.1. Motor synchrony and social connectedness

Motor synchrony can occur spontaneously [1,3,14] or be induced using exercises with a partner, such as performing arm curls, lifting fingers at a specified tempo or matching arm movements in a mirror game [12,15,16]. Both spontaneous and induced motor synchrony are reliably associated with increased prosocial behaviours and experiences of closeness [2–4], such as increased self-other overlap on questionnaire reports [17,18]. According to Shamay-Tsoory *et al*. [19], motor synchrony, or alignment, overlaps with emotional and cognitive alignment in that all three are complementary manifestations of social connectedness. Following on from this explanation, individuals who experience social difficulties are likely to engage in these forms of alignment less frequently. Indeed, reduced spontaneous motor synchrony is observed in clinical populations known to exhibit social difficulties, such as individuals diagnosed with attention-deficit/hyperactive disorder (ADHD), autism, bipolar disorder and social anxiety [20–24].

Shamay-Tsoory *et al*.'s [19] extended integrative model of alignment comprises three components: first, a gap-monitoring system, linked to dorsal anterior cingulate cortex (ACC), dorsal medial prefrontal cortex (PFC) and anterior insulae evaluates the predicted and existent alignment with a social partner (e.g. [25,26]). When a gap in alignment is detected, the alignment system (or observation–execution system) activates the inferior-frontal gyrus (IFG), inferior parietal lobule, premotor cortex and superior temporal sulcus to facilitate alignment by perceiving a behaviour and initiating the same behaviour (e.g. [27]). When no gap is detected, the reward system, associated with the ventral striatum, orbitofrontal cortex and ventral medial PFC, is activated and drives maintenance of alignment (e.g. [28]). This model posits that adults typically seek to align with social partners by default, and that the social connectedness experienced during induced motor synchrony, likely to be processed by the reward system, is socially motivating.

### 1.2. Observer–audience dynamics and cognitive performance

Awareness of an observer, or an audience, is known to change behaviour [13]. In social observation scenarios, the observed individual's behaviour may be influenced by the task and/or social dynamics between the observed individual and their audience. An early meta-analysis by Bond & Titus [29] reported that task complexity mediated the audience effect, with simple tasks resulting in improved performance, and complex tasks resulting in poorer performance under observation. In a meta-analysis demonstrating that task complexity alone could not capture the social dynamics of an audience and observed individual, Uziel [30] synthesized 14 studies centred on personality traits of the observed individual, revealing that elevated extraversion and self-esteem were associated with improved performance under observation, whereas neuroticism and low self-esteem were associated with poorer performance.

Though meta-analyses exist that summarize effect of task-type and characteristics of the observed individual, a meta-analysis of audience characteristics is yet to be curated. In the meantime, we must draw evidence from individual studies, which often do not disentangle task complexity and audience characteristics. From existing work, we learn that an attentive audience (e.g. signalled by direct gaze) enhances performance more than an inattentive or invisible audience [31]. A friendly, non-expert, peer-audience with little knowledge of the task goal can improve performance, whereas a higher status or expert audience can worsen performance if their knowledge of the task is not made explicit [31–34]. Klein *et al*. [34] propose that an audience's explicit knowledge of the goal may induce more commitment to the goal, and thereby improved performance. Further, increased rapport between a higher status, expert audience and observed individual can also improve performance [35,36]. Several studies also document that rapport, or the ease of social interaction, improves with increasing motor synchrony between individuals [37–39]. Thus, it stands to reason that induced motor synchrony between an individual and an audience—for argument's sake, a peer of the same status with no task-related expertise—has the potential to increase rapport and improve cognitive performance.

### 1.3. Enhancing inhibitory control with a motor synchrony activity

To date, only a few studies have sought to quantify changes in cognitive performance resulting from a motor synchrony activity (e.g. [9,12]). Rauchbauer *et al*. [12] report that young adult participants, whose postures were implicitly mimicked by a confederate for 20 min prior to performing an automatic imitation task, showed better inhibition than participants who were not mimicked by the confederate. Keisari *et al*. [9] investigated the influence of the mirror game on elderly individuals' cognitive performance, reporting improved working-memory span and recognition of speech in noise after elderly individuals played the mirror game relative to when they participated in a group exercise class. In both studies, the cognitive tasks were performed under the supervision of an experimenter and the synchronized partner was not in the room. These studies offer evidence that motor synchrony can enhance cognitive performance generally. Rauchbauer *et al*. [12] specifically demonstrate that inhibition of motor responses can be improved by prior motor synchrony with a peer. We further note that studies examining benefits of t'ai chi (which involves synchronous group movement) on inhibitory control in elderly and substance-addicted populations report improvement after interventions lasting several weeks [40,41].

In the present study, we examine how recent synchronization with a partner influences response inhibition, when one is observed by that same partner. This question is relevant to the need to provide therapeutic options for people with inhibition difficulties [42], including those with autism, ADHD, schizophrenia and social anxiety diagnoses [21,43–45]. In addition to reduced response inhibition, individuals with these disorders also show reduced spontaneous motor synchrony [46]. Moreover, in clinical settings, the degree of spontaneous motor synchrony with a therapist has been demonstrated to predict therapy duration and outcomes [43–45]. Spontaneous motor synchrony is also related to treatment compliance [21]. It follows, therefore, that an intervention targeting both response inhibition and motor synchrony could potentially be valuable in clinical settings.

### 1.4. Current study

This study assessed the extent to which inducing motor synchrony between an observed individual and their audience boosts the observed individual's ability to suppress motor responses (i.e. inhibitory control). This was achieved using measures of behavioural performance (reaction times and error rates) and cortical haemodynamic brain activity recorded over frontal brain regions using functional near-infrared spectroscopy (fNIRS). These are measured from and compared between a Synchronized group, in which participant–audience motor synchrony is induced via the mirror game [15], and a non-synchronized Control group, in which each member of the observed participant–audience pair takes a turn observing the other member move their arms. To obtain performance and cortical measures of inhibitory control, we employ a simple response inhibition task (Go/NoGo task) after the movement task. We selected the Go/NoGo task (described in more detail in Methods) for its relative simplicity, so that a non-threatening peer observer should have a positive influence on participants' performance (e.g. [31]).

Using fNIRS, we recorded changes in cortical oxygenation from the frontal brain regions reported to be activated by inhibitory control and observation by an audience. Functional magnetic resonance imaging (fMRI) and fNIRS studies measuring the influence of an audience on haemodynamic brain activity report increased activity in medial PFC, anterior cingulate cortex (ACC) and striatum–brain areas associated with self-monitoring and reward systems [13,47–52]. Studies investigating inhibitory control, i.e. response suppression, report increased activity in prefrontal and inferior-frontal brain regions, as well as the ACC, insulae and thalami [47,52–58]. We measured changes in cortical oxygenation as a proxy for neural activity in five regions of interest: left and right IFG, as well as left, right, and middle PFC. We did not measure from the subcortical structures mentioned above, as the penetration depth of fNIRS is approximately 1.5 cm beneath the scalp [59], and our hypotheses pertain to cortical regions involved in social processing [13].

As preregistered (https://osf.io/87xnj/), we hypothesized that both groups should respond more quickly during blocks requiring no inhibition of motor responses **(Hypothesis 1)**. We also hypothesized that the Synchronized group will respond faster than the Control group across blocks, regardless of response inhibition requirements **(Hypothesis 2)** and will fail to inhibit responses less frequently than the Control group **(Hypothesis 3)**. With respect to the changes in cortical oxygenation measured using fNIRS, we hypothesized that blocks that require response inhibition will evoke greater cortical activation than blocks that do not in right PFC (as right PFC activation is more commonly observed with fNIRS while right IFG is more commonly observed with fMRI), but not in other ROIs for both groups **(Hypothesis 4)**. Finally, we evaluated an exploratory hypothesis that the Synchronized group relative to Control group may differ between block types and/or per ROIs **(Hypothesis 5)**.

## 2. Methods

### 2.1. Participants

A total of 68 participants were recruited from Macquarie University in Sydney, Australia. All participants met the self-reported inclusion criteria being right-handed, aged 18–40, having no history of head injury, neurological or psychiatric diagnoses, and not currently taking a psycho-pharmaceutical medication (SSRIs or Ritalin). Following König *et al*. [60], we added further inclusion criteria that participants must report no alcohol consumption within the 12 h prior or tetrahydrocannabinol (THC) use/exposure within the 24 h prior to the study, and not playing videogames frequently (e.g. more than once a week, as inhibition is a skill required in many videogames, and we did not wish to recruit expert inhibitors). Of the 68 participants who met each of these initial inclusion criteria for participation, nine were excluded following data collection: Five were not deceived by the story explaining the confederate's presence, two did not perform the Go/NoGo task correctly, one reported during the session that they actually played video games frequently (after reporting they did not during initial screening), and one participant's session was interrupted by a fire alarm.

The remaining 59 participants were pseudo-randomly assigned to either the Synchronized group or non-synchronized (Control) group. To ensure a balanced sample, groups were counterbalanced for gender, age, and confederate (*n* = 2, both female, aged 21 and 30) in a continuous fashion, with additional participants recruited following exclusions. The Synchronized group consisted of 30 participants (14 female, 16 male; mean age = 22.10 ± 5.78 years) and the Control group of 29 participants (15 female, 13 male, 1 other; mean age = 21.00 ± 4.93 years). Participants' consumption of caffeine and alcohol prior to the experiment was recorded to ensure equal distribution across the two groups (electronic supplementary material, [S1]).

Ethical approval for this study was obtained from the Macquarie University Human Research Ethics Committee (Ref: 520221102239451). Written informed consent was obtained from participants before beginning the session, at which time participants were told that a confederate was a new student volunteer visiting the laboratory for the first time. Consent was renegotiated after the completion of the Go/NoGo task, when participants were given the opportunity to withdraw their data if they were not comfortable with the minor deception about the confederate. No participants withdrew their consent. Participants received course credit or a cash honorarium (AU$30) for their involvement.

### 2.2. Questionnaires

To explore whether extraversion may influence our measures of interest (reaction times, commission errors or cortical activation) and need to be included in our models, we conducted preregistered and exploratory preliminary analyses (described in §2.5). Participants completed a questionnaire based on the International Personality Item Pool (IPIP) representations of the extraversion subscale of the Goldberg [61] Big Five markers and the Rosenberg [62] self-esteem scale. Participants completed the questionnaire on their mobile phone before being welcomed to the laboratory, thereby ensuring a sense of privacy and confidentiality prior to the experiment (i.e. neither the experimenter nor confederate saw how the participant responded). The extraversion scale used a 5-point Likert scale (1 = *very inaccurate* to 5 = *very accurate*) to respond to 5 positively and 5 negatively worded items (I feel comfortable around people or I don't talk a lot). The self-esteem scale used a 4-point Likert scale (0 = *strongly disagree*, 3 = *strongly agree*) to respond to 5 positively and 5 negatively worded items (I know my strengths or I am less capable than most people). Both scales were scored by summing the points, with the points for negatively worded items reversed. In our analyses, Extraversion refers to the summed extraversion and self-esteem scores per participant.

### 2.3. Procedure

The experimenter greeted all participants, introduced the confederate as a student volunteer visiting the laboratory for the first time, and asked the participants if they would feel comfortable if the confederate observed, and participated in certain easy activities, in place of the experimenter (script available at https://osf.io/87xnj/). Participants next completed the synchronizing or control movement activity with the confederate, then afterward, completed the Go/NoGo task under the observation of the confederate.

#### 2.3.1. Synchronizing/control movement activities

Participants in the Synchronized group completed a synchronizing activity with the confederate, and participants in the Control group completed a movement observation activity with the confederate.

*Synchronized group: synchronizing activity.* The participant and the confederate were instructed that they would be playing the mirror game, where they were to mirror the other person's upper-body movements as closely as possible, that each person would take a turn as the leader for 2:30 min, and that the leader should try to vary their movements, to encourage participants to make use of the space around them. The participant was always assigned to lead the first turn and the confederate led the second.

*Control group: movement observation activity.* The participant and the confederate were instructed that they would be doing a movement activity, where each person would take a turn moving their upper body for 2:30 min while the other person observed and completed an observation task. As in the synchronizing activity, they were instructed that the person moving should try to vary their movements. Before beginning, the participant and the confederate each drew an observation task from a hat (e.g. count the number of times your partner raises their right hand above their ear). To reduce social awkwardness, the confederate always took the first turn moving their upper body, and the participant took the second turn.

The synchronizing and control activities were identical in that participants sat face-to-face, looking at each other, and engaged in movements of similar intensity across both groups. The only differences were whether the participants moved synchronously or separately and whether the participant or confederate moved first. We selected this control activity on the basis that, relative to a passive observation task, it is engaging for both parties, and relative to an anti-mirror task (i.e. moving simultaneously, but avoiding matching each other's movements), it eliminates the possibility of temporally contingent motor patterns, which are also a form of motor synchrony [63]. The duration of 2:30 min was selected on the basis that once each person took a turn leading, the 5 min duration would be consistent with recent work [15], while also maximizing the influence of this manipulation without inducing boredom.

**Figure 1.**
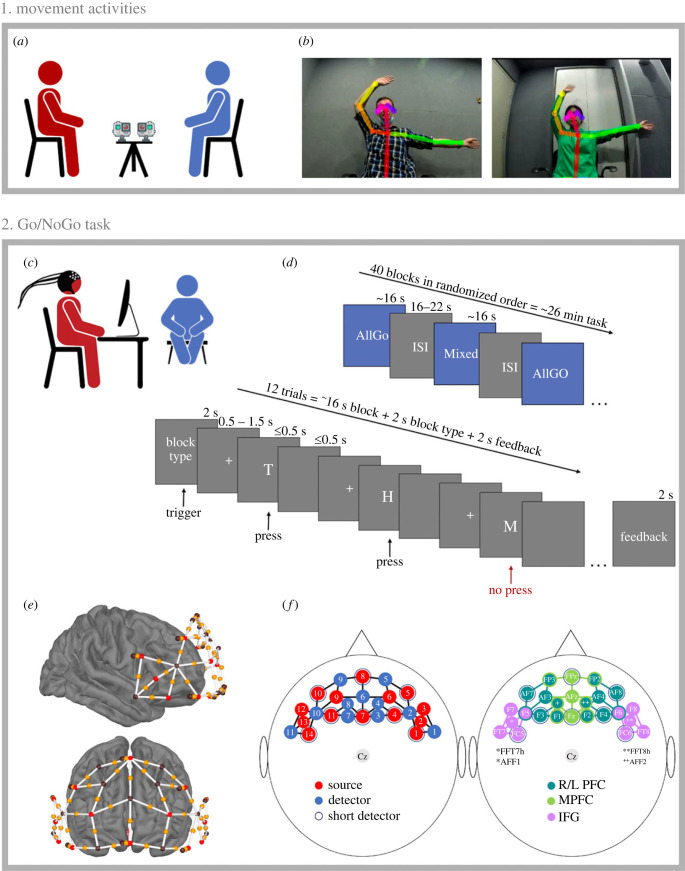
(*a*) First, participants completed either a synchronizing or control movement activity with a confederate. The activity was video-recorded using a pair of GoPros positioned between the participant and confederate dyad, who were seated for the activity. (*b*) The similarity of each dyad's movements was calculated using coordinates of each person's joints per frame, as estimated with OpenPose. (*c*) Next, the participant completed a Go/NoGo task while the confederate observed from approximately 1.5 m away, in the participant's peripheral vision. (*d*) The Go/NoGo task consisted of AllGo blocks (100% Go trials) and Mixed blocks (66% Go and 33% NoGo trials). (*e*) fNIRS recordings were made using a montage covering the inferior-frontal and prefrontal brain regions. White bars = channels between source–detector pairs; red spheres = sources; black spheres = detectors; yellow spheres = point of measurement. (*f*) 10–10 positions of source and detector optodes, as well as the channels belonging to each region of interest; enlarged version in electronic supplementary material (S1).

For these activities, the confederate and participant were seated facing each other (1.2 m apart) with a pair of GoPro HERO3+ video-cameras (GoPro, San Mateo, CA, USA) between them, one facing each person (figure 1*a,b*). Recordings were made using OBS studio (https://obsproject.com/). The experimenter attended to the recording computer in the corner of the room, approximately 2.5 m away from the participant and confederate.

#### 2.3.2. Go/NoGo task

A Go/NoGo task adapted from Young *et al*. [64] was used to obtain behavioural and cortical measures of inhibition. Each participant sat in front of a computer in a sound-shielded room and was instructed that they would see the letters T, H, N, W and M on the screen, and that they should press the space bar on the keyboard when they saw T, H, N or W, but not when they saw M. They were also instructed to respond as quickly and accurately as possible. As such T, H, N and W were presented in Go trials, and M was presented in NoGo trials. Participants completed 40 blocks of 12 trials with 20 blocks consisting of only Go trials (AllGo blocks; no Ms included) and 20 blocks consisting of 66% Go trials and 33% NoGo trials (Mixed blocks). Before each block, the type of block was presented on the screen for 2 s (‘Only T, H, N, W’ or ‘Ms included’ followed by ‘Respond as quickly and accurately as possible’). The letters were presented for up to 500 ms followed by a blank screen for up to 500 ms, allowing 1000 ms for a response (figure 1*d*). Between trials and before the first trial, a fixation cross was displayed with a jittered intertrial interval (ITI) of 500–1500 ms. Between blocks, a blank screen was shown for a jittered interstimulus interval (ISI) of 16–22 s. ITIs were jittered to avoid cyclic responding to the motor task, thereby promoting higher accuracy [65,66]. The letter stimuli and the 4 : 1 ratio of Go : NoGo stimuli (T, H, N, W : M) with 33% NoGo, as well as the ITIs of 500–1500 ms were selected to maximize both the number of commission errors (i.e. button-presses on NoGo trials; failed response inhibition) and the signal-to-noise ratio [64,67]. ISIs were jittered to reduce participants' anticipation of the onset of the upcoming block, as well as to ensure that blocks were not temporally synchronized with changes in intracranial blood pressure regulation, i.e. Mayer waves [68,69]. The experiment was programmed and presented using PsychoPy [70] and can be retrieved from https://osf.io/87xnj/.

Participants were familiarized with the task by completing one AllGo and one Mixed block before donning the fNIRS cap. Cap in place, participants completed the Go/NoGo task with no breaks in approximately 26 min with the confederate observing from approximately 1.5 m, facing the participant at a 90° angle (figure 1*c*). This was the maximal distance possible in the laboratory facilities. The 90° angle allowed the confederate to remain in the participants’ peripheral vision without inducing stress by positioning the confederate too close to the participant [31,71]. Several features of the task design were implemented to ensure that participants were aware that their performance could be observed by the confederate. First, the letters that made up the task stimuli were large enough (approx. 17 cm high) for the confederate to see from their seated position. Second, the participant's button-pressing hand was positioned in clear view for the confederate to see. Finally, the confederate was present for the presentation of task instructions, and therefore participants could safely assume the confederate knew how to evaluate the participant's performance. Unbeknownst (we assume) to the participants, the experimenter observed from an adjacent room via a video camera.

### 2.4. fNIRS equipment

#### 2.4.1. Spectrometer

fNIRS recordings were made with a NIRScoutX (NIRx Medical Technologies LLC) with 24 LED sources and 32 avalanche photodiode detectors and NIRStar software. The sources emitted wavelengths of 760 and 850 nm with a sampling rate of 4.5 Hz. The optodes were mounted onto mesh caps marked with International 10–10 positions (Easycap GmbH) using grommets and spacers to maintain a maximum 30-mm separation (NIRx Medical Technologies LLC).

#### 2.4.2. Optode positions (montage)

A montage of 14 sources, 11 detectors and 8 short detectors was used to record from bilateral and middle PFC, as well as bilateral IFG (figure 1*e,f*). To cover these brain areas, our montage comprised 38 long channels (source–detector pairs approx. 30 mm apart), along with 8 short channels (source–detector pairs 8 mm apart), distributed across the ROIs to account for location-dependent heterogeneity in the extracerebral signals [72–74]. Optode positions within the montage were determined using the AAL2 atlas in the fOLD toolbox [75–77]. To enhance the reliability of our findings, we analyse ROIs as opposed to individual channels [78], given that final positioning of optodes on participants' heads may have varied slightly between participants.

### 2.5. Manipulation check

To verify that participant–audience motor synchrony was indeed increased in the Synchronized group relative to the Control group, we quantified and compared the mean similarity of a dyad's poses—their upper-body position—in each frame of the video-recorded movement activity. This analysis was exploratory and not preregistered.

To obtain a dyad's mean pose similarity, we employed OpenPose software [79] to identify the confederate and participants' left and right wrist, elbow, shoulder and their neck in the video-recording of the movement activity (figure 1*b*). Next, we used OpenPose to estimate and write x and y coordinates, and a measure of the algorithm's confidence in these estimates between 0 and 1, per body part per person to a JSON file per frame. From here, we converted extracted JSON files for each dyad to a CSV file using a R script adapted from de Jonge-Hoekstra & Repgen (https://osf.io/6s73d/). Missing values were replaced with the median for that joint, and the timeseries for each joint was subsequently smoothed using a Savitzky–Golay filter (window length = 13 frames, polynomial order = 2) implemented with the signal R package [80]. Then, using R code adapted from Broadwell & Tangherlini [81], we estimated the Euclidian distance between all pairs of body parts for each person in a frame, storing these in a separate ‘pose matrix’ per person, and then comparing (via Laplacian procedure) the pose matrices for each frame. Pose similarity was returned as a value between 0 = *no similarity* and 1 = *identical* per frame. The mean per dyad was calculated across all frames from the video (approx. 9000 frames per video).

### 2.6. Data analysis

For our preregistered analyses, we employed a Bayesian approach to multi-level regression [82], using the brms package [83] in the R language [84] within the RStudio IDE [85]. This approach allowed us to build models incrementally [86] and to use leave-one-out cross-validation (LOO) [87] to estimate and compare the out-of-sample accuracy between simpler and more complex models. In other words, LOO informs us about the degree to which increasing complexity enhances the accuracy of our models. For key parameters in the most complex model, we report and interpret the posterior distribution with a 95% credible interval, which we calculate using the highest posterior density region (HPD) method [82]. For readers more accustomed to a Frequentist approach with *p*-values, we recommend perusing Kruschke & Liddell [88], and we offer the following (simplified) heuristic for interpreting HPDs: Comparisons can be said to entail substantial differences when HPD does not contain zero and to be trends when the tip of an HPD-tail overlaps with zero.

All models were built beginning with only varying intercepts per participant (ID) and block type where relevant. Next, simple predictors were added one at a time, followed by 2-way then 3-way interactions between predictors [86]. We used treatment coding for group (Synchronized = 1, Control = 0) and block type (AllGo = 0, Mixed = 1). We set weakly informed priors to impose a constrained distribution on our expected results, thereby acknowledging the limits of our knowledge as to our expected results, allowing for possible large effects and allowing the data to dominate the posterior distribution structure [89,90]. These priors were set using parameter values extracted from pilot data (collected using a very similar Go/NoGo task completed by 16 participants while recording fNIRS signals). Full models, comparison to simpler models, and visualization of all model parameters are reported in electronic supplementary material, (S2).

#### 2.6.1. Preliminary analysis: extraversion

We preregistered an exploratory analysis of the relationship between our measure of Extraversion and each of our data sources (reaction times, commission errors or cortical haemodynamic responses amplitudes [HbO only for this specific analysis]). Uziel's [30] meta-analysis concludes that extraversion impacts how individuals perform on cognitive tasks when observed, reporting a positive correlation between extraversion and performance. With this knowledge, we seek to determine whether including Extraversion as a predictor in our other planned analyses constitutes a parsimonious addition. The extraversion data were modelled using Gaussian regression models, with priors based on summed parameter values for extraversion and self-esteem from previous studies [91,92]. The detailed results of this analysis, as well as the models used, are reported in the electronic supplementary material, (S3). In summary, extraversion did not covary meaningfully with reaction times, commission errors or HbO amplitudes. We thus did not include Extraversion in our main analyses. However, for reaction times and HbO, some evidence of covariance with other terms (i.e. group, block type) was observed. To account for this in our models for each reaction times and cortical oxygenation, we included Extraversion as a random slope per participant.

#### 2.6.2. Go/NoGo reaction times and commission errors

To assess if the Synchronized group responded to Go trials faster than the Control group in both AllGo and Mixed blocks (Hypothesis 2), we modelled the data using lognormal models. We examined whether the Synchronized group made fewer commission errors (failed suppression of response) than the Control group (Hypothesis 3) using Poisson regression models.

#### 2.6.3. Haemodynamic response amplitude

##### 2.6.3.1. First level

Analyses were performed using MNE [93], MNE-NIRS [94] and NiLearn [95]. The generalized linear model (GLM) approach was taken to quantify the amplitude of evoked haemodynamic responses per ROI and Condition [96]. Waveforms for visual inspection are presented in the electronic supplementary material, (S4). The sampling rate of the recorded signal was reduced from 4.5 to 0.6 Hz [94]. The signal was converted from raw intensity to optical density, using absolute raw intensity values. Next, the signal was converted to concentrations of HbO and HbR using the Modified Beer–Lambert Law [97,98] with a partial pathlength factor of 0.1, accounting for both differential pathlength factor (DPF) and partial volume correction (PVC), where (DPF = 6)/(PVC = 60) is equal to 0.1 [99,100]. The GLM was fitted to the long-channel data, which was isolated by rejecting channels less than 20 mm or greater than 40 mm. The design matrix for the GLM was generated by convolving a 16-s boxcar function at each event-onset-time with the canonical haemodynamic response function [101,102]. The GLM also included all principal components of short-detector channels to account for extracerebral and physiological signal components. Further, drift orders accounting for signal components up to 0.01 Hz were included as regression factors [96]. The GLM was performed with a lag-1 autoregressive noise model, to account for the correlated nature of the fNIRS signal components. Individual coefficient estimates were then averaged for each ROI, weighted by the standard error.

##### 2.6.3.2. Second-level

To investigate whether Mixed blocks evoked greater cortical activation than AllGo blocks in right PFC only, as well as the influence of group on haemodynamic response amplitudes, we employed Bayesian multivariate Gaussian models. Fitting both HbO and HbR within the same model allows for the correlated natures of the HbO and HbR response amplitudes to inform the model fit, exploiting the available information without the risks of multicollinearity incurred by treating chromophore (i.e. HbO and HbR) as a categorical factor. Full model reported in electronic supplementary material, (S2).

For preregistered exploratory analyses, we also derived HbO–HbR difference values by subtracting HbR from HbO estimates per participant, ROI and block type. This difference measure is commonly employed in fNIRS studies addressing clinical questions [103,104] and has recently been shown to be useful in answering questions in cognitive neuroscience [105,106]. The HbO–HbR difference offers three main advantages when communicating and interpreting changes in cortical oxygenation measured with fNIRS. First, by synthesizing a pair of HbO and HbR estimates into a single value, the complexity of models and the potential for multicollinearity is strongly reduced. Second, the sign (±) of an HbO–HbR difference value is informative: positive difference values correspond to canonical haemodynamic responses, while negative values correspond to inverted responses (also called negative BOLD responses). Third, the relationship between HbO and HbR estimates can be used to categorize responses very conservatively as systemic phenomena (blood pressure changes) or true cortical responses. Here, negatively correlated HbO–HbR pairs are more likely to represent cortical activation [107], while positively correlated pairs are more likely to represent physiological confounding phenomena such as blood pressure changes, muscle oxygenation or extracerebral changes [108,109], and the latter can easily be excluded for more conservative analyses [105,106]. We present results from preregistered exploratory models fitted to negatively correlated HbO–HbR pairs here, and models fitted to all HbO–HbR pairs in electronic supplementary material, (S5). Parameter estimates from models were contrasted using the emmeans package [110].

## 3. Results

### 3.1. Manipulation check

**Figure 2.**
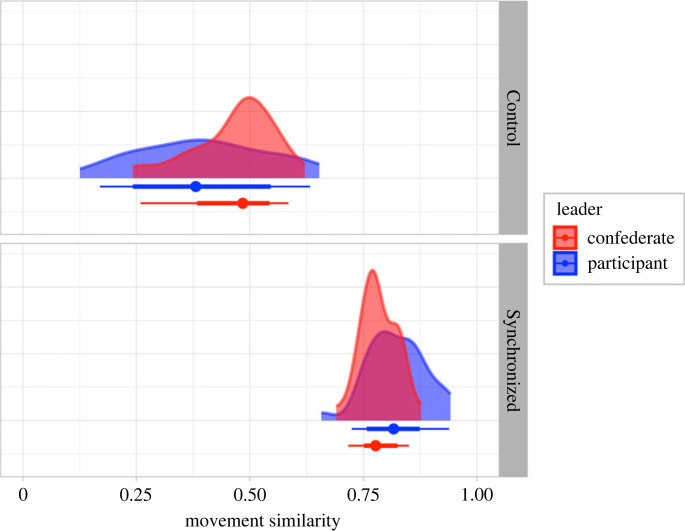
Movement similarity scores for movement activities (Synchronized group = mirror game, Control group = movement observation activity). Score calculated from body position coordinates estimated by OpenPose: 0 = *no similarity* and 1 = *identical*. Summary point shows median, bars show interval covering 66% and 95% of the raw distribution.

Before proceeding to our planned analyses, we first verified that the level of motor synchrony during the movement activities indeed differed (not preregistered). We quantified the spatial and temporal similarity of each dyads' upper-body movements, yielding a movement similarity score (0 = *no similarity* and 1 = *identical*). The mean similarity is 0.43 (s.d. = 0.12) for the Control group and 0.80 (s.d. = 0.06) for the Synchronized group, with a difference between means of 0.37. Figure 2 illustrates this substantial difference between groups and further demonstrates that similarity scores are not influenced by who is leading the mirror game (i.e. participant or confederate). One might expect values closer to zero in the Control group, however, during the movement observation activity, both members of the dyad keep their torsos and heads relatively still. This, in itself, is a form of motor synchrony, explaining why the Control group mean similarity is substantially above zero.

### 3.2. Reaction times and commission errors

**Figure 3.**
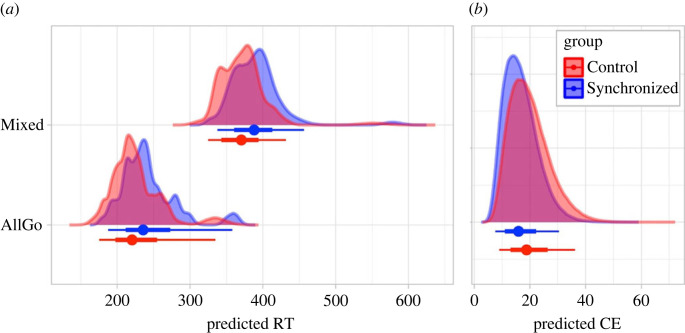
Predicted posterior distributions for (*a*) reaction times (RT) and (*b*) commission errors (CE) per group. Summary point shows median, bars show interval covering 66% and 95% of the raw distribution.

To obtain a proxy for inhibitory control, we recorded the reaction times in blocks with only Go trials requiring button-presses (AllGo) and those additionally requiring participants to inhibit motor responses in one third of trials (Mixed), and commission errors (the number of presses on NoGo trials). Our preregistered hypotheses were that (**1**) both groups would respond faster to the Go trials in AllGo than Mixed blocks, (**2**) the watchful eye of a peer, with whom one has recently synchronized, relative to a non-synchronized peer would result in faster reaction times for both block types and (**3**) fewer commission errors. To address these hypotheses, we fitted the model *RT*∼1 + Group*BlockType + (1 + BlockType|ID). Confirming our first hypothesis, reaction times were faster for AllGo than Mixed blocks (*β* = 146.8 ms, 95% highest posterior density region: HPD = [144.5, 149.1]) when both groups were considered together (figure 3). Contrary to our second hypothesis, the Control group responded faster than the Synchronized group in both block types (*β* = 18.1 ms, HPD = [15.8, 20.4]). No interaction between group and block type was predicted or observed.

Next, we examined commission errors using the model *CE*∼*1 + Group + (1 | ID)*. Consistent with our third hypothesis, the Synchronized group made fewer commission errors than the Control group (*β* = 2.93 errors, HPD = [0.75, 5.06], or converted to error rate: *β* = 3.66%, HPD = [0.94, 6.33]). Exploratory, not preregistered, analyses revealed a negative relationship, albeit small, between reaction times and commission errors (*ß* = −0.005, HPD = [−0.009, −0.002]), which suggests that for every 200 ms slowing of the response time, participants make one commission error fewer (see electronic supplementary material, S7 for comparison to findings from preregistered models). Further exploratory, not preregistered, analyses revealed no relationship between mean movement similarity per dyad and reaction times, or between mean movement similarity and commission errors in the Synchronized group (*ß* = −0.06, HPD = [−1.01, 0.87]). We observed a trend toward fewer commission errors with greater mean movement similarity in the Control group (*ß* = 0.31, HPD = [−0.38, 1.06]; electronic supplementary material, S7). No analyses of changes in motor synchrony over the course of the movement activity and behavioural measures of performance were considered, as these tasks were completed one after another rather than at the same time.

### 3.3. Cortical haemodynamic activity

**Figure 4.**
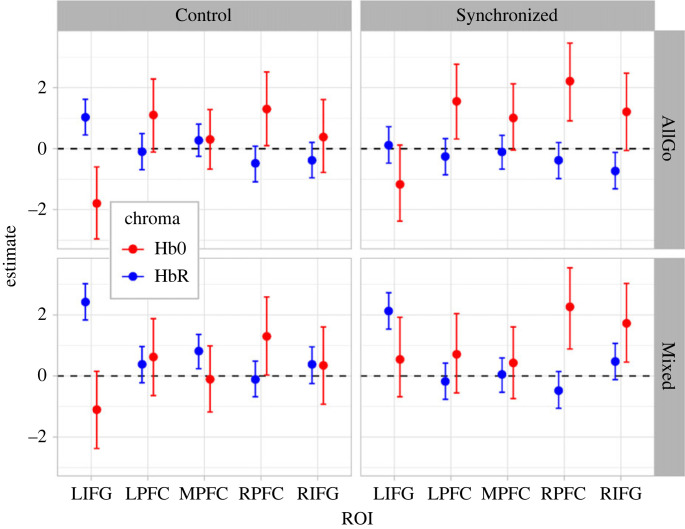
HbO and HbO parameter estimates (*ß*) per ROI, group and block type with 95% highest posterior density (HPD) region. Error bars show 95% HPD regions.

We next examined changes in cortical oxygenation evoked by inhibiting motor responses, and the influence of a peer-audience, with whom participants recently synchronized, employing the multivariate model: *(HbO, HbR)*∼1 + BlockType * ROI * Group + (1 + BlockType|p|ID) to obtain the parameter estimates in figure 3 and table 2 (note: *p* in this formula links the random effects structure to each of the outcome variables [HbO, HbR]). Our preregistered hypotheses were that (**4**) Mixed compared to AllGo blocks would evoke an enhanced haemodynamic response only in right PFC for both groups, and (**5**) proposed the exploratory analysis of group differences in either ROIs and/or block types. Counter to our fourth hypothesis, contrasts comparing Mixed and AllGo blocks with Control and Synchronized groups combined revealed no difference in right PFC. In fact, right PFC exhibited the smallest difference between block types of all ROIs for HbO (table 1 and figure 4). Further, left IFG for HbO trends toward a more positive parameter estimate for Mixed than AllGo blocks. Substantial evidence for this same pattern is observed in bilateral IFG for HbR. Subsequent contrasts addressing group differences—our fifth, exploratory, hypothesis—revealed a substantial difference in bilateral IFG for HbO only (table 3), wherein a greater difference between block types was observed in the Synchronized than Control group. The Synchronized, relative to Control, group showed substantially more positive HbO estimates in bilateral IFG for both Mixed blocks and a more negative estimate in middle PFC. In Mixed blocks, the positive HbO estimates obtained for the Synchronized group are accompanied by positive HbR estimates (table 3). Of note, these simultaneous increases in HbO and HbR in bilateral IFG did not accord with the increase in HbO and decrease in HbR expected of cortical activity. To delve further into this pattern, which suggests systemic rather than cortical changes in the signal, we exploit the strengths of the HbO–HbR difference as a derived measure synthesizing changes in concentration of HbO and HbR.

**Table 1.** Descriptive statistics (mean and standard deviation [s.d.]) for commission errors and reaction times.

	group	block type	mean	s.d.
commission errors				
	Control	Mixed	19.70	8.81
	Synchronized	Mixed	16.70	7.57
reaction times (ms)				
	Control	AllGo	230.00	23.50
	Synchronized	AllGo	247.00	46.00
	Control	Mixed	367.00	26.10
	Synchronized	Mixed	384.00	42.40

**Table 2.** AllGo-Mixed contrast estimates per ROI for HbO, HbR and negatively correlated HbO–HbR difference. Positive = AllGo block haemodynamic response estimate more positive, negative = Mixed block more positive. Substantial differences (i.e. HPD does not contain 0) marked in bold, trends (i.e. 0 in tail of HPD) marked in italics. *β* = estimate; HPD = 95% highest posterior density region.

ROI	HBO		HbR		HbO–HbR difference	
	*β*	HPD	*β*	HPD	*β*	HPD
LIFG	*−1*.*21*	*[−2.51, 0.06]*	**−1.71**	**[−2.31, −1.11]**	1.02	[−0.77, 2.91]
LPFC	0.66	[−0.67, 1.93]	−0.28	[−0.91, 0.30]	0.71	[−1.10, 2.53]
MPFC	0.49	[−0.64, 1.56]	−0.35	[−0.94, 0.21]	*1*.*28*	*[−0.07, 2.63]*
RPFC	−0.03	[−1.32, 1.29]	−0.14	[−0.75, 0.45]	−0.26	[−2.00, 1.39]
RIFG	−0.25	[−1.50, 1.07]	**−0.99**	**[−1.59, −0.39]**	0.85	[−0.99, 2.75]

**Table 3.** Control-Synchronized contrast estimates per block type and ROI for HbO, HbR and negatively correlated HbO–HbR difference. Positive = Control group haemodynamic response estimate more positive, negative = Synchronized group more positive. Substantial group differences (i.e. HPD does not contain 0) marked in bold, trends (i.e. 0 in tail of HPD) marked in italics. *β* = estimate; HPD = 95% highest posterior density region.

ROI	block type	HbO		HbR		HbO–HbR difference	
		*β*	HPD	*β*	HPD	*β*	HPD
LIFG	AllGo	−0.63	[−2.27, 0.98]	**0.92**	**[0.08, 1.71]**	**−3.34**	**[−5.48, −1.07]**
LPFC	AllGo	−0.45	[−2.05, 1.17]	0.16	[−0.67, 0.96]	−0.57	[−2.69, 1.43]
MPFC	AllGo	−0.70	[−1.94, 0.52]	0.38	[−0.31, 1.09]	*−1*.*47*	*[−3.02, 0.11]*
RPFC	AllGo	−0.92	[−2.55, 0.72]	−0.10	[−0.90, 0.72]	−0.54	[−2.58, 1.39]
RIFG	AllGo	−0.82	[−2.52, 0.71]	0.35	[−0.47, 1.16]	−1.02	[−3.10, 1.08]
LIFG	Mixed	***−*1.65**	**[−3.39, −0.06]**	0.29	[−0.56, 1.11]	**−3.93**	**[−6.40, −1.57]**
LPFC	Mixed	−0.08	[−1.78, 1.71]	0.56	[−0.30, 1.37]	−0.66	[−3.26, 1.97]
MPFC	Mixed	−0.54	[−1.98, 0.89]	*0*.*76*	*[−0.01, 1.52]*	**−1.89**	**[−3.68, −0.11]**
RPFC	Mixed	−0.96	[−2.71, 0.82]	0.37	[−0.46, 1.20]	−1.03	[−3.39, 1.24]
RIFG	Mixed	*−1*.*37*	*[−3.11, 0.40]*	−0.10	[−0.94, 0.74]	*−2*.*09*	*[−4.68, 0.50]*

### 3.4. HbO–HbR difference

**Figure 5.**
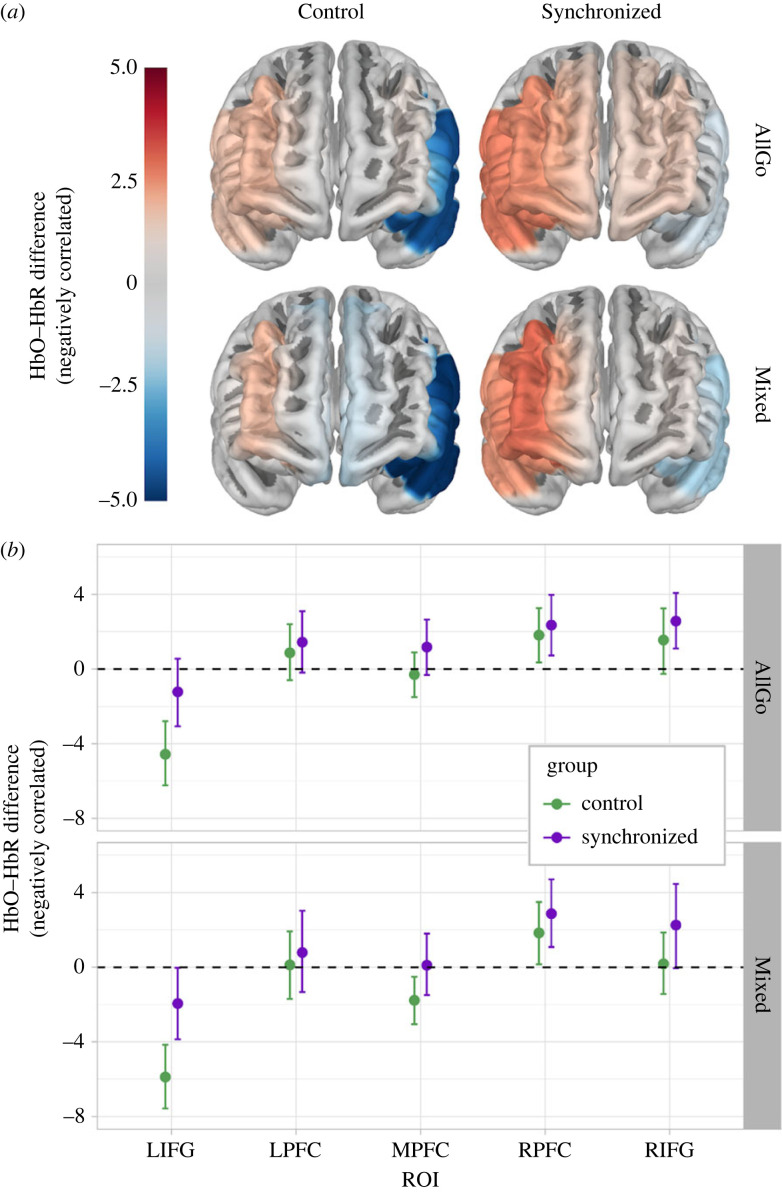
HbO–HbR difference estimates per ROI, group and block type (*a*) projected onto cortical surface and (*b*) with error bars showing 95% highest posterior density (HPD) region.

Our attention was caught by the simultaneous increase in HbO and HbR in bilateral IFG when comparing differences between Control and Synchronized groups’ cortical activity during Mixed blocks. This simultaneous increase in both HbO and HbR could plausibly reflect a physiological response, such as blood pressure changes, muscle oxygenation or extracerebral changes [108,109], evoked by the participants' anticipation of NoGo trials in the Mixed block. To isolate changes in cortical activity from the plausibly task-induced systemic responses, we proceeded to fit the model *Hbo*–*HbR.difference*∼1 + BlockType * ROI * Group + (1 + BlockType|ID) to HbO–HbR to difference values for all difference values, and subsequently, to difference values from negatively correlated HbO–HbR estimate pairs only. Here, we report estimates from the negatively correlated difference values (figure 5), taking a conservative approach that excludes systemic responses not eliminated in the first-level analysis. For comparison of estimates for models with all and negatively correlated values, refer to electronic supplementary material, (S5).

Following our planned analysis of HbO and HbR (figure 4), we applied the same post hoc contrasts as for HbO and HbR individually, and again did not find the hypothesized **(4)** difference between AllGo-Mixed blocks in right PFC with groups combined but did observe some evidence for greater middle PFC activity in the Synchronized group (table 2). Contrasts between groups for each ROI and block type **(5)** indicated larger differences between groups for Mixed than AllGo blocks, whereby the Synchronized group shows greater activation than the Control group in left IFG and middle PFC during AllGo blocks, as well as bilateral IFG and middle PFC during Mixed blocks (table 3).

#### 3.4.1. Linking brain and behaviour

Having established that a speed–accuracy trade-off is induced under the observation of a peer, with whom one has recently synchronized, we sought to explore whether these behavioural outcomes are associated with participants’ cortical activity (via exploratory, not preregistered analyses). We assessed the relationship between cortical activity and each reaction times and commission errors using the model: *HbO*–*HbR.difference*∼1 + BlockType * ROI * Group * ReactionTime * CommissionErrors + (1 + BlockTypeID). In Mixed blocks, the Synchronized group exhibited substantially greater HbO–HbR differences, i.e. greater cortical activation, with increasing reaction times in bilateral IFG and middle PFC, with left PFC showing a congruent trend (table 4). For AllGo blocks, the Synchronized group showed greater HbO–HbR difference in bilateral IFG with slowing reaction times, although the evidence is stronger for right than left IFG. The Control group showed some evidence for reduced HbO–HbR differences in right PFC for AllGo blocks and increased HbO–HbR differences in right IFG for Mixed blocks, as reaction times increased. A greater number of commission errors in Mixed blocks is associated with reductions in HbO–HbR differences in bilateral IFG for the Synchronized group, which were more substantial in left than right IFG. The Control group also shows the decreasing HbO–HbR difference in bilateral IFG with increasing commission errors, though more evidence is found for this association in right than left IFG.

**Table 4.** Associations between behavioural measures and cortical activity as observed in HbO–HbR difference (negatively correlated HbO–HbR pairs only). Substantial associations (i.e. HPD does not contain 0) marked in bold, trends (i.e. 0 in tail of HPD) marked in italics. *β* = estimate; HPD = 95% highest posterior density region.

ROI	block type	group	reaction times		commission errors	
			*β*	HPD	*β*	HPD
LIFG	AllGo	Control	−0.01	[−0.09, 0.08]	−0.26	[−0.94, 0.53]
		Synchronized	*0*.*08*	*[−0.01, 0.17]*	0.06	[−0.41, 0.53]
	Mixed	Control	0.05	[−0.04, 0.14]	*−0*.*20*	*[−0.45, 0.04]*
		**Synchronized**	**0.13**	**[0.04, 0.21]**	**−0.53**	**[−0.91, −0.14]**
LPFC	AllGo	**Control**	**−0.09**	**[−0.16, −0.01]**	−0.44	[−1.08, 0.17]
		Synchronized	0.04	[−0.02, 0.12]	0.29	[−0.12, 0.70]
	Mixed	Control	−0.01	[−0.09, 0.07]	0.01	[−0.27, 0.26]
		Synchronized	*0*.*11*	*[−0.01, 0.22]*	−0.27	[−0.77, 0.23]
MPFC	AllGo	Control	−0.03	[−0.10, 0.04]	−0.16	[−0.74, 0.45]
		Synchronized	0.04	[−0.03, 0.11]	0.11	[−0.34, 0.58]
	Mixed	Control	0.01	[−0.05, 0.07]	−0.03	[−0.29, 0.20]
		**Synchronized**	**0.08**	**[0.00, 0.17]**	−0.23	[−0.66, 0.22]
RPFC	AllGo	Control	*−0*.*07*	*[−0.15, 0.01]*	−0.34	[−1.00, 0.30]
		Synchronized	0.00	[−0.05, 0.05]	−0.01	[−0.40, 0.38]
	Mixed	Control	−0.03	[−0.11, 0.05]	0.07	[−0.17, 0.30]
		Synchronized	0.04	[−0.05, 0.12]	−0.03	[−0.46, 0.42]
RIFG	AllGo	Control	−0.01	[−0.11, 0.09]	−0.14	[−0.94, 0.64]
		**Synchronized**	**0.09**	**[0.05, 0.14]**	0.23	[−0.12, 0.60]
	Mixed	Control	*0*.*06*	*[−0.01, 0.14]*	**−0.29**	**[−0.53, −0.04]**
		**Synchronized**	**0.12**	**[0.00, 0.24]**	*−0*.*44*	*[−1.00, 0.10]*

### 3.5. Additional analyses

As per our preregistered preliminary analyses, we also fitted exploratory models for each reaction times and cortical oxygenation to assess whether including random slopes of Extraversion scores per participant explained the data better. Visual inspection of data split per confederate for each measure (electronic supplementary material, [S2]) led to further exploratory models for commission errors and cortical oxygenation with random coefficients (slopes for a categorical variable) of Confederate per participant. We also fitted an exploratory model for cortical oxygenation with random slopes for movement similarity across participants to account for individual differences in achieved motor synchrony. None of these exploratory models offered substantially better out-of-sample predictions (electronic supplementary material, [S2]), meaning that the addition of each given variable did not contribute meaningfully to the model, and does not influence the outcome variable (i.e. reaction times or cortical oxygenation).

## 4. Discussion

Combining behavioural and cortical measures, we examined the influence of an audience with whom one has synchronized versus a non-synchronized audience on inhibitory control using a relatively simple Go/NoGo task. We found evidence for differential engagement of IFG across the synchronized and non-synchonized conditions when participants made fewer commission errors. Specifically, when participants were observed by a peer without prior synchronization, making fewer commission errors was associated with increased activity in right IFG, indicative of response inhibition. However, participants observed by a peer after synchonization demonstrated slower reaction times and fewer commission errors, resulting in increased activity in bilateral IFG, suggesting the involvement of cognitive processes beyond response inhibition (table 4). Middle and left PFC activity increased with increasing reaction times only when the observed individual and audience had completed a synchronizing activity together. These findings demonstrate that the watchful eye of a synchronized peer incurs a speed–accuracy trade-off, accompanied by stronger activation of bilateral IFG, as well as left and middle PFC.

### 4.1. The presence of an audience results in improved accuracy at the expense of speed

The presence of an audience is widely reported to improve cognitive performance by helping the observed individual ignore task-irrelevant information, yielding faster and more accurate responses [9,12,13,29,31]. Recent evidence also suggests that a synchronizing activity can improve cognitive performance [9–12] by enhancing self-monitoring processes [12,111], in addition to enhancing social connectedness, affiliation, feelings of closeness and self-other overlap [2,16–19]. Although we did not measure affiliation enhancement, feelings of closeness or self-other overlap in the current study, it seems reasonable to assume that our Synchronized group experienced these manifestations of social connectedness, based on the reliability of these effects across previous studies [2,19] and our rigorous quantification of each dyad's movement similarity during the mirror game or movement observation task (figure 2). Further, we ensured that our participants had never met the confederates prior to the experiment, and that all sessions were run following the same script, maximizing the likelihood that any change in perceived closeness within each dyad, over the course of the session, was a direct result of the synchronizing mirror game or movement observation activity. However, it is important to consider that differences in the overall amount of movement or solo versus joint movements might contribute to our findings (this limitation is discussed further below).

We hypothesized that the presence of a recently synchronized observer could improve performance on a Go/NoGo task more than a non-synchronized observer in terms of both speed and accuracy (hypotheses 2 and 3). Our data demonstrated that the presence of a recently synchronized observer did indeed boost accuracy, but at the cost of speed. The trade-off is small (i.e. 3.66% fewer commission errors for 18.10 ms slower responses), but greater than trade-offs previously induced using non-social rewards [112]. Padmala & Pessoa [112] suggest that the trade-off results from non-social reward-based motivation, which incurs greater self-monitoring, much like motor synchrony. Motor synchrony also incurs both reward processing and self-monitoring [12,19,111]. As such, we attribute the speed–accuracy trade-off that occurs in the presence of the recently synchronized audience to greater behavioural motivation, which likely stems from stronger social alignment induced by the synchronizing activity [19]. Moreover, maintaining social alignment requires continuous monitoring for gaps in alignment [19], and this continuous process may interfere with reaction speeds, resulting in slower reaction times. In the light of the slower reaction times observed in this study, we propose that maintaining social alignment may outweigh the cost of slight reductions in behavioural performance [113]. The findings from our analyses of changes in cortical oxygenation offer additional insight into the neural mechanisms supporting response inhibition under observation by an audience with whom one has recently synchronized.

### 4.2. Right IFG indexes more than inhibition in the presence of an audience

Inhibition of motor responses has been pinpointed to right IFG using fMRI [47,52–55,58]. fNIRS studies have more consistently measured functional responses to tasks requiring inhibitory control, such as the Go/NoGo task used here, in right PFC [56,57,114,115]. This difference may be related to the coarser spatial resolution of approximately 2–3 cm of fNIRS, compared to 3 mm in fMRI [59,116]. As we used fNIRS, our preregistered hypothesis (4) was that we would observe increased right PFC activity during Mixed, relative to AllGo, blocks. This expected difference did not manifest in either group (table 2 and figure 5), nor was this difference present in right IFG when contrasting block types. The lack of a difference does not categorically imply that right IFG and PFC are inactive during the Go/NoGo task: In fact, substantial activation is observed in both right IFG and PFC during both block types for the Synchronized group, with the Control group showing activation in both right IFG and PFC for AllGo blocks, and only in right IFG for Mixed blocks.

Turning to our next hypothesis (5), that we might observe different patterns of cortical activation between groups, we delved further into right IFG activation in our exploratory analyses, revealing that slower reaction times and fewer commission errors correlated with greater cortical activity in right IFG in both groups (table 4). The slope estimates for the Synchronized, relative to Control, group were greater for both behavioural measures, suggesting that right IFG may index processes related to the presence of the audience, in concert with inhibition itself. However, additional comparisons to performance without the presence of the audience would be needed to confirm this. It is plausible that the by-products of motor synchrony, including increased perceived closeness and self-other overlap, may drive the stronger association between behaviour and right IFG activity, as well as the speed–accuracy trade-off. Reaction times were strongly linked with cortical activation in right IFG for both block types, while commission errors were more closely linked with right IFG activity during Mixed than AllGo blocks (i.e. greater uncertainty for the latter). However, this difference between block types is not meaningful for the Synchronized group (table 4), meaning that the positive association between slower, more accurate, responses and right IFG activity is unlikely to index increased inhibition *per se*. Instead, this difference may index increased attentional mechanisms [58,112], self-monitoring [117] and/or perceived closeness [118] related to maintenance of social alignment with the synchronized audience [19]. This final possibility is consistent with emerging findings from hyperscanning research, suggesting that shared right IFG activity is indicative of interpersonal coupling within interacting dyads [119,120].

### 4.3. A recently synchronized audience increases self-monitoring

Previous fMRI studies investigating the neural correlates of an audience's presence report increased haemodynamic activity in the dorsomedial prefrontal cortex and striatum [48–50]. These regions—which lie beyond the penetration depth of fNIRS—are known to be engaged in mentalizing processes [121] and encode social network information associated with relationship value [122,123]. They also support self-monitoring and motivation [47,52,124] and are the purported generators of the ‘medial frontal negativities', electrophysiological responses indexing error monitoring and feedback integration [124,125]. These negativities have been demonstrated to be greater when an individual is observed by a friend, when compared with a stranger [126,127], and when observed by peers of a similar age, relative to older peers [128]. It is thus proposed that these electrophysiological responses index the perceived closeness between the observed individual and the audience. Kang *et al*. [127] propose that self-other overlap may mediate this relationship, while Ferguson *et al*. [128] posit in-group/out-group dynamics as a potential explanation. These findings from different modalities can be further enriched by Shamay-Tsoory *et al*.'s [19] proposition that the ACC and medial PFC monitor for gaps in social alignment; another form of self-monitoring.

Middle, and to a lesser extent, left PFC activity and reaction times were correlated for the Synchronized group only, whereby greater activation was observed for slower response times. Dorsomedial frontal activity has previously been reported to increase under social observation, purportedly serving the functional role of regulating of behavioural motivation [48–50]. From this perspective, individuals in the Synchronized group who responded more slowly and accurately may have experienced a greater degree of behavioural motivation because of their motor synchrony with their audience. Alternatively, the increased middle PFC activation could index the sustained self-monitoring required to ensure continued social alignment with the synchronized audience [19]. Another possible explanation comes from Hester *et al*. [129], who reported that individuals who responded more slowly on a Go/NoGo task showed greater midline activity and higher self-reported absent-mindedness scores. Individuals who responded more slowly may have been mildly distracted by the presence of their recently synchronized partner. This explanation is improbable, as we did not observe reduced accuracy as well as slower response times. Considering our findings and the proliferation of research corroborating that both an audience and motor synchrony can improve cognitive performance, we propose that the association between reaction times and middle PFC activity observed in the Synchronized group, and not the Control group, points to increased behavioural motivation and/or increased self-monitoring to maintain social alignment with the audience.

In the Control group, we observed a group-level inverted BOLD response in left IFG during Mixed blocks (figure 5), as well as a trend wherein individuals who sacrificed accuracy for speed exhibited stronger inverted BOLD responses (table 4). The Synchronized group showed the same association in left IFG for reaction times, but with steeper slopes, relative to the Control group, for both Mixed and AllGo blocks. Closer examination showed a greater number of inverted BOLD responses in left IFG for Mixed blocks for Control group (84.61% of negatively correlated HbO–HbR pairs) than for the Synchronized group (70.37%), which were also more pronounced in the Control group (figure 5). Padmala & Pessoa [112] also report inverted BOLD responses in left IFG, which lessen in amplitude with the introduction of non-social reward-based motivation. From this, we interpret that social motivation induced by an observer may potentially be analogous to reward motivation and functionally reduce the amplitude of inverted BOLD responses in a similar fashion. We also observed enhancement of left IFG activity with increasing errors for the Synchronized group during AllGo blocks: This may add nuance to the explanation above in that perhaps the enhanced social motivation experienced by the Synchronized group was lessened during the AllGo blocks but remained constant across both block types for the Control group.

### 4.4. Implications for clinicians and future directions

These findings suggest that synchronizing activities have the potential to improve self-monitoring while inhibiting motor responses. This could be particularly beneficial for individuals who have known difficulties in response inhibition and spontaneous mirroring, which has been documented in several psychological, neurodevelopmental and psychiatric conditions such as ADHD, social anxiety, autism and schizophrenia [21,43–46]. For instance, a prior synchronizing activity between the clinician and patient has potential to improve supervised response inhibition. Further research undertaken in clinical setting is needed and is supported by mounting evidence that response inhibition can be improved through training [130–133]. However, further research is also needed to understand the influence of a dyad's social relationship (i.e. peers, strangers, patient–clinician, parent–child) on response inhibition in the presence of an audience.

In addition to exploring the dynamics of varying social relationships, future studies should further consider including measures relating to the change in perceived closeness and self-other overlap to verify whether these factors mediate the increased attentional mechanism and self-monitoring that appear to underpin the speed–accuracy trade-off reported here. To do this, a ‘free-movement’ control condition could be added. Here, both members of the dyad could move at the same time, but not in synchrony and without any temporal contingencies, while the experimenter is in another room. Ideally, the overall amount of movement would be accounted for during analysis. Together, these methodological improvements could help disentangle the effects of motor synchrony *per se* from the overall amount of movement and the experience of joint movement versus solo movement, which may also contribute to the group-level differences observed in the present study. Another control condition to consider in future research would involve the synchronizing activity followed by the response inhibition task with *no* audience/observer. This condition could be compared to the two conditions used in the present study to gain insight into the relative contributions of the audience effect and the synchronizing activity in improving response inhibition.

An open question remains regarding the length of time any benefits of synchonization last. In the present study, there was approximately 20 min between participants engaging in the synchronizing activity and completing the Go/NoGo task, while the fNIRS cap was donned with the peer-audience observing. The Go/NoGo task then lasted approximately 15 min. Together, this suggests that the effects synchonizing lasted at least 35 min, with the uninterrupted presence of the synchronized observer. Further empirical interrogation is required to ascertain whether the influence of a synchronized observer lasts longer than 35 min and whether interruptions in the interaction reduce the observed effect. Moreover, future research could also delve into the relationship between the degree of motor synchrony per dyad and Go/NoGo performance, during the task itself, as this might yield more fine-grained insight into the mechanisms through which motor synchrony impacts cognition.

Further insight into the network underpinnings of the speed–accuracy trade-off could also be gained from examining connectivity within frontal brain regions [57,134]. The inclusion of additional brain regions in such connectivity analyses, including the temporal parietal junction, inferior parietal lobule and premotor areas involved in maintenance of motor, and more generally, social alignment [19,135–138] would also be beneficial. Further, concurrent recording of neural activity (i.e. hyperscanning using fNIRS) from both the observed individual engaging in response inhibition and a genuinely interested audience, such as a clinician, could also provide valuable insight into the socially mediated cognitive processes discussed here [19,139,140].

## 5. Conclusion

This study demonstrated that the presence of a recently synchronized peer-audience can improve accuracy on a Go/NoGo task probing inhibitory control, at the cost of reaction speed. Further, this study demonstrated that increased cortical activity in bilateral IFG and middle PFC measured using fNIRS was associated with slower reaction times and fewer errors in the presence of a partner with whom one has recently synchronized. We propose that this relationship reflects increased self-monitoring that helps maintain social alignment.

## Data Availability

The data and all code used to run experiment, as well as the preregistration and parameters for the cleaning and analysis of the data described in this study are available at https://osf.io/87xnj/ [141]. Supplementary material is available online [142].

## References

[1] Lakin JL, Jefferis VE, Cheng CM, Chartrand TL. 2003 The chameleon effect as social glue: evidence for the evolutionary significance of nonconscious mimicry. J. Nonverb. Behav. **27**, 145-162. (10.1023/A:1025389814290)

[2] Mogan R, Fischer R, Bulbulia JA. 2017 To be in synchrony or not? A meta-analysis of synchrony's effects on behavior, perception, cognition and affect. J. Exp. Soc. Psychol. **72**, 13-20. (10.1016/j.jesp.2017.03.009)

[3] Rennung M, Göritz AS. 2016 Prosocial consequences of interpersonal synchrony: a meta-analysis. Zeitschr. Psychol. **224**, 168. (10.1027/2151-2604/a000252) PMC5137339 28105388

[4] Vicaria IM, Dickens L. 2016 Meta-analyses of the intra- and interpersonal outcomes of interpersonal coordination. J. Nonverb. Behav. **40**, 335-361. (10.1007/s10919-016-0238-8)

[5] Koehne S, Behrends A, Fairhurst MT, Dziobek I. 2016 Fostering social cognition through an imitation-and synchronization-based dance/movement intervention in adults with autism spectrum disorder: a controlled proof-of-concept study. Psychother. Psychosom. **85**, 27-35. (10.1159/000441111) 26609704

[6] Landa RJ, Holman KC, O'Neill AH, Stuart EA. 2011 Intervention targeting development of socially synchronous engagement in toddlers with autism spectrum disorder: a randomized controlled trial: RCT of social intervention for toddlers with ASD. J. Child Psychol. Psych. **52**, 13-21. (10.1111/j.1469-7610.2010.02288.x) PMC3059234 21126245

[7] Morris P, Hope E, Foulsham T, Mills JP. 2021 The effectiveness of mirroring- and rhythm-based interventions for children with autism spectrum disorder: a systematic review. Rev. J. Autism Dev. Disorders **8**, 541-561. (10.1007/s40489-021-00236-z)

[8] Srinivasan SM, Kaur M, Park IK, Gifford TD, Marsh KL, Bhat AN. 2015 The effects of rhythm and robotic interventions on the imitation/praxis, interpersonal synchrony, and motor performance of children with autism spectrum disorder (ASD): a pilot randomized controlled trial. Autism Res. Treatment **2015**, 1-18. (10.1155/2015/736516) PMC4697072 26793394

[9] Keisari S, Feniger-Schaal R, Palgi Y, Golland Y, Gesser-Edelsburg A, Ben-David B. 2020 Synchrony in old age: playing the mirror game improves cognitive performance. Clin. Gerontol. **45**, 312-326. (10.1080/07317115.2020.1799131) 32762289

[10] Nahardiya G, Markus A, Bennet R, Shamay-Tsoory SG. 2022 The benefits of learning movement sequences in social interactions. Front. Psychol. **13**, 901900. (10.3389/fpsyg.2022.901900) 36017441 PMC9396234

[11] Pärnamets P, Espinosa L, Olsson A. 2020 Physiological synchrony predicts observational threat learning in humans. Proc. R. Soc. B **287**, 20192779. (10.1098/rspb.2019.2779) PMC7287361 32429814

[12] Rauchbauer B, Dunbar RIM, Lamm C. 2020 Being mimicked affects inhibitory mechanisms of imitation. Acta Psychol. **209**, 103132. (10.1016/j.actpsy.2020.103132) 32683097

[13] Hamilton A, Lind F. 2016 Audience effects: What can they tell us about social neuroscience, theory of mind and autism? Cult. Brain **4**, 159-177. (10.1007/s40167-016-0044-5) 27867833 PMC5095155

[14] Sebanz N, Bekkering H, Knoblich G. 2006 Joint action: Bodies and minds moving together. Trends Cogn. Sci. **10**, 70-76. (10.1016/j.tics.2005.12.009) 16406326

[15] Feniger-Schaal R, Schönherr D, Altmann U, Strauss B. 2021 Movement synchrony in the mirror game. J. Nonverb. Behav. **45**, 107-126. (10.1007/s10919-020-00341-3)

[16] Ravreby I, Shilat Y, Yeshurun Y. 2022 Liking as a balance between synchronization, complexity and novelty. Sci. Rep. **12**, 3181. (10.1038/s41598-022-06610-z) 35210459 PMC8873358

[17] Miles LK, Nind LK, Henderson Z, Macrae CN. 2010 Moving memories: behavioral synchrony and memory for self and others. J. Exp. Soc. Psychol. **46**, 457-460. (10.1016/j.jesp.2009.12.006)

[18] Paladino M-P, Mazzurega M, Pavani F, Schubert TW. 2010 Synchronous multisensory stimulation blurs self-other boundaries. Psychol. Sci. **21**, 1202-1207. (10.1177/0956797610379234) 20679523

[19] Shamay-Tsoory SG, Saporta N, Marton-Alper IZ, Gvirts HZ. 2019 Herding brains: a core neural mechanism for social alignment. Trends Cogn. Sci. **23**, 174-186. (10.1016/j.tics.2019.01.002) 30679099

[20] Asher M, Kauffmann A, Aderka IM. 2020 Out of sync: nonverbal synchrony in social anxiety disorder. Clin. Psychol. Sci. **8**, 280-294. (10.1177/2167702619894566)

[21] Dean DJ, Scott J, Park S. 2021 Interpersonal coordination in schizophrenia: a scoping review of the literature. Schizophr. Bull. **47**, 1544-1556. (10.1093/schbul/sbab072) 34132344 PMC8530389

[22] Fitzpatrick P, Romero V, Amaral JL, Duncan A, Barnard H, Richardson MJ, Schmidt RC. 2017 Social motor synchronization: insights for understanding social behavior in autism. J. Autism Dev. Disord. **47**, 2092-2107. (10.1007/s10803-017-3124-2) 28425022

[23] Kupper Z, Ramseyer F, Hoffmann H, Tschacher W. 2015 Nonverbal synchrony in social interactions of patients with schizophrenia indicates socio-communicative deficits. PLoS ONE **10**, e0145882. (10.1371/journal.pone.0145882) 26716444 PMC4696745

[24] Zimmermann R, Fürer L, Kleinbub JR, Ramseyer FT, Hütten R, Steppan M, Schmeck K. 2021 Movement synchrony in the psychotherapy of adolescents with borderline personality pathology – a dyadic trait marker for resilience? Front. Psychol. **12**, 660516. (10.3389/fpsyg.2021.660516) 34276484 PMC8277930

[25] Cacioppo S, Zhou H, Monteleone G, Majka EA, Quinn KA, Ball AB, Norman GJ, Semin GR, Cacioppo JT. 2014 You are in sync with me: neural correlates of interpersonal synchrony with a partner. Neuroscience **277**, 842-858. (10.1016/j.neuroscience.2014.07.051) 25088911

[26] Fairhurst MT, Janata P, Keller PE. 2013 Being and feeling in sync with an adaptive virtual partner: brain mechanisms underlying dynamic cooperativity. Cereb. Cortex **23**, 2592-2600. (10.1093/cercor/bhs243) 22892422

[27] Iacoboni M. 2009 Neurobiology of imitation. Curr. Opin Neurobiol. **19**, 661-665. (10.1016/j.conb.2009.09.008) 19896362

[28] Knutson B, Cooper JC. 2005 Functional magnetic resonance imaging of reward prediction. Curr. Opin Neurol. **18**, 411-417. (10.1097/01.wco.0000173463.24758.f6) 16003117

[29] Bond CF, Titus LJ. 1983 Social facilitation: a meta-analysis of 241 studies. Psychol. Bull. **94**, 265-292. (10.1037/0033-2909.94.2.265) 6356198

[30] Uziel L. 2007 Individual differences in the social facilitation effect: A review and meta-analysis. J. Res. Personality **41**, 579-601. (10.1016/j.jrp.2006.06.008)

[31] Huguet P, Galvaing MP, Monteil JM, Dumas F. 1999 Social presence effects in the Stroop task: further evidence for an attentional view of social facilitation. J. Pers. Soc. Psychol. **77**, 1011-1025. (10.1037/0022-3514.77.5.1011) 10573878

[32] Belletier C, Davranche K, Tellier IS, Dumas F, Vidal F, Hasbroucq T, Huguet P. 2015 Choking under monitoring pressure: being watched by the experimenter reduces executive attention. Psychon. Bull. Rev. **22**, 1410-1416. (10.3758/s13423-015-0804-9) 25673216

[33] Eastvold AD, Belanger HG, Vanderploeg RD. 2012 Does a third party observer affect neuropsychological test performance? It depends. Clin. Neuropsychol. **26**, 520-541. (10.1080/13854046.2012.663000) 22420506

[34] Klein HJ, Lount RB, Park HM, Linford BJ. 2020 When goals are known: the effects of audience relative status on goal commitment and performance. J. Appl. Psychol. **105**, 372-389. (10.1037/apl0000441) 31414830

[35] Barnett MD, Parsons TD, Reynolds BL, Bedford LA. 2018 Impact of rapport on neuropsychological test performance. Appl. Neuropsychol. Adult **25**, 258-265. (10.1080/23279095.2017.1293671) 28631989

[36] Barnett MD, Sawyer J, Moore J. 2022 An experimental investigation of the impact of rapport on Stroop test performance. Appl. Neuropsychol. Adult **29**, 941-945. (10.1080/23279095.2020.1828081) 33032451

[37] Bernieri FJ. 1988 Coordinated movement and rapport in teacher-student interactions. J. Nonverb. Behav. **12**, 120-138. (10.1007/BF00986930)

[38] Miles LK, Nind LK, Macrae CN. 2009 The rhythm of rapport: interpersonal synchrony and social perception. J. Exp. Soc. Psychol. **5**, 585-589. (10.1016/j.jesp.2009.02.002)

[39] Sharon-David H, Mizrahi M, Rinott M, Golland Y, Birnbaum GE. 2019 Being on the same wavelength: Behavioral synchrony between partners and its influence on the experience of intimacy. J. Soc. Personal Relation. **36**, 2983-3008. (10.1177/0265407518809478)

[40] Menglu S, Ruiwen L, Suyong Y, Dong Z. 2021 Effects of Tai Chi on the executive function and physical fitness of female methamphetamine dependents: a randomized controlled trial. Front. Psychiatry **12**, 653229. (10.3389/fpsyt.2021.653229) 34177646 PMC8222617

[41] Yang Y, Chen T, Shao M, Yan S, Yue GH, Jiang C. 2020 Effects of Tai Chi Chuan on inhibitory control in elderly women: an fNIRS study. Front. Hum. Neurosci. **13**, 476. (10.3389/fnhum.2019.00476) 32038205 PMC6988574

[42] Gordon J. 2017 An experimental therapeutic approach to psychosocial interventions. See https://www.nimh.nih.gov/about/director/messages/2017/an-experimental-therapeutic-approach-to-psychosocial-interventions.

[43] Altmann U, Schoenherr D, Paulick J, Deisenhofer A-K, Schwartz B, Rubel JA, Stangier U, Lutz W, Strauss B. 2020 Associations between movement synchrony and outcome in patients with social anxiety disorder: evidence for treatment specific effects. Psychother. Res. **30**, 574-590. (10.1080/10503307.2019.1630779) 31213149

[44] Ramseyer F, Tschacher W. 2011 Nonverbal synchrony in psychotherapy: coordinated body movement reflects relationship quality and outcome. J. Consult. Clin. Psychol. **79**, 284-295. (10.1037/a0023419) 21639608

[45] Reinecke KCH, Joraschky P, Lausberg H. 2022 Hand movements that change during psychotherapy and their relation to therapeutic outcome: an analysis of individual and simultaneous movements. Psychother. Res. **32**, 104-114. (10.1080/10503307.2021.1925989) 33975526

[46] Wright L, Lipszyc J, Dupuis A, Thayapararajah SW, Schachar R. 2014 Response inhibition and psychopathology: a meta-analysis of go/no-go task performance. J. Abnorm. Psychol. **123**, 429-439. (10.1037/a0036295) 24731074

[47] Chevrier AD, Noseworthy MD, Schachar R. 2007 Dissociation of response inhibition and performance monitoring in the stop signal task using event-related fMRI. Hum. Brain Mapp. **28**, 1347-1358. (10.1002/hbm.20355) 17274022 PMC6871417

[48] Chib VS, Adachi R, O'Doherty JP. 2018 Neural substrates of social facilitation effects on incentive-based performance. Soc. Cogn. Affect. Neurosci. **13**, 391-403. (10.1093/scan/nsy024) 29648653 PMC5928408

[49] Finger EC, Marsh AA, Kamel N, Mitchell DGV, Blair JR. 2006 Caught in the act: the impact of audience on the neural response to morally and socially inappropriate behavior. Neuroimage **33**, 414-421. (10.1016/j.neuroimage.2006.06.011) 16891125

[50] Izuma K, Saito DN, Sadato N. 2010 Processing of the incentive for social approval in the ventral striatum during charitable donation. J. Cogn. Neurosci. **22**, 621-631. (10.1162/jocn.2009.21228) 19320552

[51] Somerville LH, Jones RM, Ruberry EJ, Dyke JP, Glover G, Casey BJ. 2013 The medial prefrontal cortex and the emergence of self-conscious emotion in adolescence. Psychol. Sci. **24**, 1554-1562. (10.1177/0956797613475633) 23804962 PMC3742683

[52] Zhang R, Geng X, Lee TMC. 2017 Large-scale functional neural network correlates of response inhibition: an fMRI meta-analysis. Brain Struct. Funct. **222**, 3973-3990. (10.1007/s00429-017-1443-x) 28551777 PMC5686258

[53] Aron AR, Robbins TW, Poldrack RA. 2004 Inhibition and the right inferior frontal cortex. Trends Cogn. Sci. **8**, 170-177. (10.1016/j.tics.2004.02.010) 15050513

[54] Cai W, Ryali S, Chen T, Li C-SR, Menon V. 2014 Dissociable roles of right inferior frontal cortex and anterior insula in inhibitory control: evidence from intrinsic and task-related functional parcellation, connectivity, and response profile analyses across multiple datasets. J. Neurosci. **34**, 14 652-14 667. (10.1523/JNEUROSCI.3048-14.2014) PMC4212065 25355218

[55] Gavazzi G, Giovannelli F, Currò T, Mascalchi M, Viggiano MP. 2021 Contiguity of proactive and reactive inhibitory brain areas: a cognitive model based on ALE meta-analyses. Brain Imag Behav. **15**, 2199-2214. (10.1007/s11682-020-00369-5) PMC8413163 32748318

[56] Kaga Y et al. 2020 Executive dysfunction in medication-naïve children with ADHD: a multi-modal fNIRS and EEG study. Brain Dev. **42**, 555-563. (10.1016/j.braindev.2020.05.007) 32532641

[57] Nguyen T, Condy EE, Park S, Friedman BH, Gandjbakhche A. 2021 Comparison of functional connectivity in the prefrontal cortex during a simple and an emotional go/no-go task in female versus male groups: an fnirs study. Brain Sci. **11**, 909. (10.3390/brainsci11070909) 34356143 PMC8304823

[58] Schulz KP, Clerkin SM, Halperin JM, Newcorn JH, Tang CY, Fan J. 2009 Dissociable neural effects of stimulus valence and preceding context during the inhibition of responses to emotional faces. Hum. Brain Mapp. **30**, 2821-2833. (10.1002/hbm.20706) 19086020 PMC2733937

[59] Pinti P, Tachtsidis I, Hamilton A, Hirsch J, Aichelburg C, Gilbert S, Burgess PW. 2020 The present and future use of functional near-infrared spectroscopy (fNIRS) for cognitive neuroscience. Ann. N Y Acad. Sci. **1464**, 5-29. (10.1111/nyas.13948) 30085354 PMC6367070

[60] König N, Steber S, Borowski A, Bliem H, Rossi S. 2021 Neural processing of cognitive control in an emotionally neutral context in anxiety patients. Brain Sci. **11**, 543. (10.3390/brainsci11050543) 33925958 PMC8146407

[61] Goldberg L. 1992 The development of markers for the Big-Five factor structure. Psychol. Assess. **4**, 26-42. (10.1037/1040-3590.4.1.26)

[62] Rosenberg M. 1965 Society and the adolescent self-image. Princeton, NJ: Princeton University Press.

[63] Rauchbauer B, Grosbras M-H. 2020 Developmental trajectory of interpersonal motor alignment: Positive social effects and link to social cognition. Neurosci. Biobehav. Rev. **118**, 411-425. (10.1016/j.neubiorev.2020.07.032) 32783968 PMC7415214

[64] Young ME, Sutherland SC, McCoy AW. 2018 Optimal go/no-go ratios to maximize false alarms. Behav. Res. Methods **50**, 1020-1029. (10.3758/s13428-017-0923-5) 28664243

[65] Lee RWY, Jacobson LA, Pritchard AE, Ryan MS, Yu Q, Denckla MB, Mostofsky S, Mahone EM. 2015 Jitter reduces response-time variability in ADHD: an ex-Gaussian analysis. J. Atten. Disord. **19**, 794-804. (10.1177/1087054712464269) 23190614 PMC3600392

[66] Wodka EL, Simmonds DJ, Mahone EM, Mostofsky SH. 2009 Moderate variability in stimulus presentation improves motor response control. J. Clin. Exp. Neuropsychol. **31**, 483-488. (10.1080/13803390802272036) 18686112 PMC2892264

[67] Wessel JR. 2018 Prepotent motor activity and inhibitory control demands in different variants of the go/no-go paradigm. Psychophysiology **55**, e12871. (10.1111/psyp.12871) 28390090

[68] Julien C. 2006 The enigma of Mayer waves: facts and models. Cardiovasc. Res. **70**, 12-21. (10.1016/j.cardiores.2005.11.008) 16360130

[69] Luke R, Shader MJ, McAlpine D. 2021 Characterization of Mayer-wave oscillations in functional near-infrared spectroscopy using a physiologically informed model of the neural power spectra. Neurophotonics **8**, 041001. (10.1117/1.NPh.8.4.041001) 34901310 PMC8652350

[70] Peirce JW, Hirst RJ, MacAskill MR. 2022 Building experiments in PsychoPy, 2nd edn. London, UK: Sage.

[71] Bogdanova OV, Bogdanov VB, Dureux A, Farnè A, Hadj-Bouziane F. 2021 The peripersonal space in a social world. Cortex **142**, 28-46. (10.1016/j.cortex.2021.05.005) 34174722

[72] Brigadoi S, Cooper RJ. 2015 How short is short? Optimum source–detector distance for short-separation channels in functional near-infrared spectroscopy. Neurophotonics **2**, 025005. (10.1117/1.nph.2.2.025005) 26158009 PMC4478880

[73] Gagnon L, Cooper RJ, Yücel MA, Perdue KL, Greve DN, Boas DA. 2012 Short separation channel location impacts the performance of short channel regression in NIRS. Neuroimage **59**, 2518-2528. (10.1016/j.neuroimage.2011.08.095) 21945793 PMC3254723

[74] Zhang Y, Tan F, Xu X, Duan L, Liu H, Tian F, Zhu C-Z. 2015 Multiregional functional near-infrared spectroscopy reveals globally symmetrical and frequency-specific patterns of superficial interference. Biomed. Optics Express **6**, 2786. (10.1364/boe.6.002786) PMC4541508 26309744

[75] Rolls ET, Joliot M, Tzourio-Mazoyer N. 2015 Implementation of a new parcellation of the orbitofrontal cortex in the automated anatomical labeling atlas. Neuroimage **122**, 1-5. (10.1016/j.neuroimage.2015.07.075) 26241684

[76] Tzourio-Mazoyer N, Landeau B, Papathanassiou D, Crivello F, Etard O, Delcroix N, Mazoyer B, Joliot M. 2002 Automated anatomical labeling of activations in SPM using a macroscopic anatomical parcellation of the MNI MRI single-subject brain. Neuroimage **15**, 273-289. (10.1006/nimg.2001.0978) 11771995

[77] Morais Z, Balardin GA, Sato JB, R J. 2018 FNIRS Optodes' Location Decider (fOLD): a toolbox for probe arrangement guided by brain regions-of-interest. Sci. Rep. **8**, 1-11. (10.1038/s41598-018-21716-z) 29463928 PMC5820343

[78] Wiggins IM, Anderson CA, Kitterick PT, Hartley DEH. 2016 Speech-evoked activation in adult temporal cortex measured using functional near-infrared spectroscopy (fNIRS): Are the measurements reliable? Hear. Res. **339**, 142-154. (10.1016/j.heares.2016.07.007) 27451015 PMC5026156

[79] Cao Z, Hidalgo G, Simon T, Wei SE, Sheikh Y. 2019 Openpose: Realtime multi-person 2D pose estimation using part affinity fields. See http://arxiv.org/abs/1812.08008 10.1109/TPAMI.2019.2929257 31331883

[80] signal developers. 2014 *signal: Signal processing* [Computer software]. See http://r-forge.r-project.org/projects/signal/

[81] Broadwell P, Tangherlini TR. 2021 Comparative K-Pop choreography analysis through deep-learning pose estimation across a large video corpus. Digital Human. Quarterly **15**.

[82] McElreath R. 2020 Statistical rethinking; a Bayesian course with examples in R and Stan, 2nd edn. New York, NY: Chapman & Hall.

[83] Bürkner P-C. 2017 brms: an R package for Bayesian multilevel models using Stan. J. Stat. Softw. **80**, 1-28. (10.18637/jss.v080.i01)

[84] R Core Team. 2022 R: a language and environment for statistical computing [computer software]. Vienna, Austria: R Foundation for Statistical Computing.

[85] RStudio Team. 2020 RStudio: integrated development for R [computer software]. Boston, MA: RStudio.

[86] Barr DJ. 2013 Random effects structure for testing interactions in linear mixed-effects models. Front. Psychol. **4**, 3-4. (10.3389/fpsyg.2013.00328) 23761778 PMC3672519

[87] Vehtari A, Gelman A, Gabry J. 2017 Practical Bayesian model evaluation using leave-one-out cross-validation and WAIC. Statist. Comput. **27**, 1413-1432. (10.1007/s11222-016-9696-4)

[88] Kruschke JK, Liddell TM. 2018 The Bayesian New Statistics: hypothesis testing, estimation, meta-analysis, and power analysis from a Bayesian perspective. Psychon. Bull. Rev. **25**, 178-206. (10.3758/s13423-016-1221-4) 28176294

[89] Gelman A. 2006 Prior distributions for variance parameters in hierarchical models (comment on article by Browne and Draper). Bayesian Anal. **1**, 515-534. (10.1214/06-BA117A)

[90] Lemoine NP. 2019 Moving beyond noninformative priors: Why and how to choose weakly informative priors in Bayesian analyses. Oikos **128**, 912-928. (10.1111/oik.05985)

[91] Guenole N, Chernyshenko OS. 2005 The suitability of Goldberg's big five IPIP personality markers in New Zealand: a dimensionality, bias, and criterion validity evaluation. New Zealand J. Psychol. **34**, 86-96.

[92] Sinclair SJ, Blais MA, Gansler DA, Sandberg E, Bistis K, LoCicero A. 2010 Psychometric properties of the rosenberg self-esteem scale: overall and across demographic groups living within the United States. Eval Health Prof **33**, 56-80. (10.1177/0163278709356187) 20164106

[93] Gramfort A et al. 2013 MEG and EEG data analysis with MNE-Python. Front. Neurosci. **7**, 1-13. (10.3389/fnins.2013.00267) 24431986 PMC3872725

[94] Luke R, Larson E, Shader MJ, Innes-Brown H, Van Yper L, Lee AKC, Sowman PF, McAlpine D. 2021 Analysis methods for measuring passive auditory fNIRS responses generated by a block-design paradigm. Neurophotonics **8**, 025008. (10.1117/1.nph.8.2.025008) 34036117 PMC8140612

[95] Abraham A, Pedregosa F, Eickenberg M, Gervais P, Mueller A, Kossaifi J, Gramfort A, Thirion B, Varoquaux G. 2014 Machine learning for neuroimaging with scikit-learn. Front. Neuroinform. **8**, 14. (10.3389/fninf.2014.00014) 24600388 PMC3930868

[96] Huppert TJ. 2016 Commentary on the statistical properties of noise and its implication on general linear models in functional near-infrared spectroscopy. Neurophotonics **3**, 010401. (10.1117/1.nph.3.1.010401) 26989756 PMC4773699

[97] Delpy DT, Cope M, Van Der Zee P, Arridge S, Wray S, Wyatt J. 1988 Estimation of optical pathlength through tissue from direct time of flight measurement. Phys. Med. Biol. **33**, 1433-1442. (10.1088/0031-9155/33/12/008) 3237772

[98] Kocsis L, Herman P, Eke A. 2006 The modified Beer-Lambert law revisited. Phys. Med. Biol. **51**, N91-N98. (10.1088/0031-9155/51/5/N02) 16481677

[99] Santosa H, Zhai X, Fishburn F, Huppert TJ. 2018 The NIRS Brain AnalyzIR Toolbox. Algorithms **11**, 73. (10.3390/a11050073) PMC11218834 38957522

[100] Strangman GE, Franceschini MA, Boas DA. 2003 Factors affecting the accuracy of near-infrared spectroscopy concentration calculations for focal changes in oxygenation parameters. Neuroimage **18**, 865-879. (10.1016/S1053-8119(03)00021-1) 12725763

[101] Glover GH. 1999 Deconvolution of impulse response in event-related BOLD fMRI. Neuroimage **9**, 416-429. (10.1006/nimg.1998.0419) 10191170

[102] Santosa H, Huppert TJ, Fishburn F, Zhai X. 2019 Investigation of the sensitivity-specificity of canonical- and deconvolution-based linear models in evoked functional near-infrared spectroscopy. Neurophotonics **6**, 1-10. (10.1117/1.NPh.6.2.025009) PMC6541797 31172019

[103] Kaynezhad P et al. 2019 Quantification of the severity of hypoxic-ischemic brain injury in a neonatal preclinical model using measurements of cytochrome-c-oxidase from a miniature broadband-near-infrared spectroscopy system. Neurophotonics **6**, 1. (10.1117/1.NPh.6.4.045009) PMC6855218 31737744

[104] Kolyva C, Ghosh A, Tachtsidis I, Highton D, Cooper CE, Smith M, Elwell CE. 2014 Cytochrome c oxidase response to changes in cerebral oxygen delivery in the adult brain shows higher brain-specificity than haemoglobin. Neuroimage **85**, 234-244. (10.1016/j.neuroimage.2013.05.070) 23707584 PMC3898943

[105] Moffat R, Başkent D, Luke R, McAlpine D, Van Yper L. 2023 Cortical haemodynamic responses predict individual ability to recognise vocal emotions with uninformative pitch cues but do not distinguish different emotions. Hum. Brain Mapp. **44**, 3684-3705. (10.1002/hbm.26305) 37162212 PMC10203806

[106] Moffat R, Cross ES. 2023 *Awareness of embodiment maximises enjoyment and engages sensorimotor cortices*. See https://osf.io/preprints/psyarxiv/y5s89.

[107] Wolf M, Wolf U, Toronov V, Michalos A, Paunescu LA, Choi JH, Gratton E. 2002 Different time evolution of oxyhemoglobin and deoxyhemoglobin concentration changes in the visual and motor cortices during functional stimulation: a near-infrared spectroscopy study. Neuroimage **16**, 704-712. (10.1006/nimg.2002.1128) 12169254

[108] Yücel MA et al. 2021 Best practices for fNIRS publications. Neurophotonics **8**, 1-34. (10.1117/1.nph.8.1.012101) PMC7793571 33442557

[109] Zimeo Morais GA, Scholkmann F, Balardin JB, Furucho RA, de Paula RCV, Biazoli CE, Sato JR. 2017 Non-neuronal evoked and spontaneous hemodynamic changes in the anterior temporal region of the human head may lead to misinterpretations of functional near-infrared spectroscopy signals. Neurophotonics **5**, 1. (10.1117/1.NPh.5.1.011002) PMC5566266 28840166

[110] Lenth RV. 2021 *emmeans: Estimated marginal means, aka least-squares means* [Computer software].

[111] Zimmermann M, Lomoriello AS, Konvalinka I. 2022 Intra-individual behavioural and neural signatures of audience effects and interactions in a mirror-game paradigm. R. Soc. Open Sci. **9**, 211352. (10.1098/rsos.211352) 35223056 PMC8847899

[112] Padmala S, Pessoa L. 2010 Interactions between cognition and motivation during response inhibition. Neuropsychologia **48**, 558-565. (10.1016/j.neuropsychologia.2009.10.017) 19879281 PMC2813998

[113] Kampis D, Southgate V. 2020 Altercentric cognition: How others influence our cognitive processing. Trends Cogn. Sci. **24**, 945-959. (10.1016/j.tics.2020.09.003) 32981846

[114] Ishii S, Kaga Y, Tando T, Aoyagi K, Sano F, Kanemura H, Sugita K, Aihara M. 2017 Disinhibition in children with attention-deficit/hyperactivity disorder: Changes in [oxy-Hb] on near-infrared spectroscopy during ‘rock, paper, scissors' task. Brain Dev. **39**, 395-402. (10.1016/j.braindev.2016.12.005) 28094161

[115] Monden Y et al. 2015 Individual classification of ADHD children by right prefrontal hemodynamic responses during a go/no-go task as assessed by fNIRS. NeuroImage: Clin. **9**, 1-12. (10.1016/j.nicl.2015.06.011) 26266096 PMC4528046

[116] Goense J, Bohraus Y, Logothetis NK. 2016 fMRI at high spatial resolution: implications for BOLD-Models. Front. Comput. Neurosci. **10**, 66. (10.3389/fncom.2016.00066) 27445782 PMC4923185

[117] Parthimos TP, Karavasilis E, Rankin KP, Seimenis I, Leftherioti K, Papanicolaou AC, Miller B, Papageorgiou SG, Papatriantafyllou JD. 2019 The neural correlates of impaired self-monitoring among individuals with neurodegenerative dementias. J. Neuropsychiatry Clin. Neurosci. **31**, 201-209. (10.1176/appi.neuropsych.17120349) 30605361

[118] Parkinson C, Liu S, Wheatley T. 2014 A common cortical metric for spatial, temporal, and social distance. J. Neurosci. **34**, 1979-1987. (10.1523/JNEUROSCI.2159-13.2014) 24478377 PMC6827593

[119] Czeszumski A, Eustergerling S, Lang A, Menrath D, Gerstenberger M, Schuberth S, Schreiber F, Rendon ZZ, König P. 2020 Hyperscanning: a valid method to study neural inter-brain underpinnings of social interaction. Front. Hum. Neurosci. **14**, 39. (10.3389/fnhum.2020.00039) 32180710 PMC7059252

[120] Minagawa Y, Xu M, Morimoto S. 2018 Toward interactive social neuroscience: neuroimaging real-world interactions in various populations. Jap. Psychol. Res. **60**, 196-224. (10.1111/jpr.12207)

[121] Frith CD, Frith U. 2006 The neural basis of mentalizing. Neuron **50**, 531-534. (10.1016/j.neuron.2006.05.001) 16701204

[122] Krienen FM, Tu P-C, Buckner RL. 2010 Clan mentality: evidence that the medial prefrontal cortex responds to close others. J. Neurosci. **30**, 13 906-13 915. (10.1523/JNEUROSCI.2180-10.2010) 20943931 PMC2989424

[123] Parkinson C, Kleinbaum AM, Wheatley T. 2017 Spontaneous neural encoding of social network position. Nat. Hum. Behav. **1**, 0072. (10.1038/s41562-017-0072)

[124] van Noordt SJR, Segalowitz SJ. 2012 Performance monitoring and the medial prefrontal cortex: a review of individual differences and context effects as a window on self-regulation. Front. Hum. Neurosci. **6**, 197. (10.3389/fnhum.2012.00197) 22798949 PMC3394443

[125] Ullsperger M, von Cramon DY. 2001 Subprocesses of performance monitoring: a dissociation of error processing and response competition revealed by event-related fMRI and ERPs. Neuroimage **14**, 1387-1401. (10.1006/nimg.2001.0935) 11707094

[126] He Q, Sun Q, Shi Z, Zhang X, Hu F. 2018 Effect of social distance on outcome evaluation in self-other decision-making: evidence from event-related potentials. Neuroreport **29**, 1499-1503. (10.1097/WNR.0000000000001141) 30303858

[127] Kang SK, Hirsh JB, Chasteen AL. 2010 Your mistakes are mine: self-other overlap predicts neural response to observed errors. J. Exp. Soc. Psychol. **46**, 229-232. (10.1016/j.jesp.2009.09.012)

[128] Ferguson HJ, Brunsdon VEA, Bradford EEF. 2018 Age of avatar modulates the altercentric bias in a visual perspective-taking task: ERP and behavioral evidence. Cogn. Affect. Behav. Neurosci. **18**, 1298-1319. (10.3758/s13415-018-0641-1) 30242574 PMC6244738

[129] Hester R, Fassbender C, Garavan H. 2004 Individual differences in error processing: a review and reanalysis of three event-related fMRI studies using the GO/NOGO task. Cereb. Cortex **14**, 986-994. (10.1093/cercor/bhh059) 15115734

[130] Allom V, Mullan B, Hagger M. 2016 Does inhibitory control training improve health behaviour? A meta-analysis. Health Psychol. Rev. **10**, 168-186. (10.1080/17437199.2015.1051078) 26058688

[131] Hartmann L, Sallard E, Spierer L. 2016 Enhancing frontal top-down inhibitory control with Go/NoGo training. Brain Struct. Funct. **221**, 3835-3842. (10.1007/s00429-015-1131-7) 26459141

[132] Meyer KN, Santillana R, Miller B, Clapp W, Way M, Bridgman-Goines K, Sheridan MA. 2020 Computer-based inhibitory control training in children with Attention-Deficit/Hyperactivity Disorder (ADHD): evidence for behavioral and neural impact. PLoS ONE **15**, e0241352. (10.1371/journal.pone.0241352) 33253237 PMC7703966

[133] Schroder E, Dubuson M, Dousset C, Mortier E, Kornreich C, Campanella S. 2020 Training inhibitory control induced robust neural changes when behavior is affected: a follow-up study using cognitive event-related potentials. Clin. EEG Neurosci. **51**, 303-316. (10.1177/1550059419895146) 31858835

[134] Ayaz H et al. 2022 Optical imaging and spectroscopy for the study of the human brain: status report. Neurophotonics **9**, S24001. (10.1117/1.NPh.9.S2.S24001) 36052058 PMC9424749

[135] Bardi L, Six P, Brass M. 2017 Repetitive TMS of the temporo-parietal junction disrupts participant's expectations in a spontaneous Theory of Mind task. Soc. Cogn. Affect. Neurosci. **12**, 1775-1782. (10.1093/scan/nsx109) 28981914 PMC5691804

[136] Sowden S, Catmur C. 2015 The role of the right temporoparietal junction in the control of imitation. Cereb. Cortex **25**, 1107-1113. (10.1093/cercor/bht306) 24177989 PMC4380005

[137] Spengler S, von Cramon DY, Brass M. 2009 Control of shared representations relies on key processes involved in mental state attribution. Hum. Brain Mapp. **30**, 3704-3718. (10.1002/hbm.20800) 19517530 PMC6870802

[138] Spengler S, Brass M, Kühn S, Schütz-Bosbach S. 2010 Minimizing motor mimicry by myself: self-focus enhances online action-control mechanisms during motor contagion. Conscious Cogn. **19**, 98-106. (10.1016/j.concog.2009.12.014) 20116291

[139] Hamilton A. 2021 Hyperscanning: beyond the hype. Neuron **109**, 404-407. (10.1016/j.neuron.2020.11.008) 33259804

[140] Moffat R, Casale CE, Cross ES. 2024 Mobile fNIRS for exploring inter-brain synchrony across generations and time. Front. Neuroergonomics **4**, 1-9. (10.3389/fnrgo.2023.1260738) PMC10790948 38234472

[141] Moffat R, Caruana N, Cross ES. 2024 Improving inhibition with motor synchrony. OSF. (https://osf.io/87xnj/)

[142] Moffat R, Caruana N, Cross ES. 2024 Inhibiting responses under the watch of a recently synchronised peer increases self-monitoring: evidence from functional near-infrared spectroscopy. Figshare. (10.6084/m9.figshare.c.7065773)38378138

